# Supplementary material for: Impact of the National Vaccination Strategy on the Prevalence of Streptococcus pneumoniae and Its Serotypes Among Clinically Healthy Children Under Six Years of Age During and After the COVID-19 Pandemic
Source: Vaccines (Basel). 2025 Jun 12;13(6):634. doi: 10.3390/vaccines13060634 (PMC12197779; doi:10.3390/vaccines13060634)
Supplement: Supplementary file 1 [file vaccines-13-00634-s001.zip › vaccines-3654217-supplementary.pdf]

Supplementary Table S1. Sequence of primers and probes used in real-time PCR for detection of Streptococcus pneumoniae to the lytA gene. Thermal conditions for conducting the PCR reaction.

**Pathogen**	**Gene**	**Primer and Probe**	**Sequence**	nM
Streptococcus pneumoniae	*lytA*	F	ACGCAATCTAGCAGATGAAGCA	**250**
		R	TCGTGCGTTTTAATTCCAGCT	**250**
		P	HEX–TGCCGAAAACGCTTGATACAGGGAG	**100**
**PCR Conditions**				
**Step**	**Cycles**	**Temperature**	**Time**	
1. Initial denaturation	1	95°C	2 min.	
2.1 Denaturation	40	95°C	15 sec.	
2.2 Annealing		55°C	30 sec.	
